# Chromosome-level genome assembly of a kombucha isolate of *Kregervanrija fluxuum*

**DOI:** 10.1128/mra.00992-26

**Published:** 2026-10-01

**Authors:** Steve A. James, Nerice Ryan, David J. Baker, Arjan Narbad

**Affiliations:** 1 Food, Microbiome and Health Programme, Quadram Institute Bioscience, Norwich, United Kingdom; 2 Centre for Microbial Interactions, Quadram Institute Bioscience 7308, Norwich, United Kingdom; 3 School of Biological Sciences, University of East Anglia 6106, Norwich, United Kingdom; Rochester Institute of Technology, Rochester, New York, USA

**Keywords:** kombucha, fungi, yeasts, *Kregervanrija fluxuum*, genome sequence

## Abstract

*Kregervanrija fluxuum* is a non-fermentative yeast and member of the *Kregervanrija* clade in the family Pichiaceae. Here, we report the draft genome sequence of *K. fluxuum* NCYC 4492, a kombucha-derived isolate.

## ANNOUNCEMENT

*Kregervanrija fluxuum* (syn. *Candida vini*) is an ascomycetous yeast often isolated from fermented beverages and vegetable brines (1). Typically recovered as a film former growing on air-exposed beer and wine, *K. fluxuum* can be found in kombucha (1, 2). Here, we combined short- and long-read sequencing to assemble the genome sequence of *K. fluxuum* NCYC 4492, isolated from a bottle of Norfolk-produced organic kombucha purchased in February 2024 in Norwich, UK. A 50 μL kombucha sample was plated onto yeast malt (YM) agar (10 g/L glucose, 3 g/L malt extract, 5 g/L peptone, and 3 g/L yeast extract) supplemented with penicillin (25 U/mL) and streptomycin (25 U/mL) and incubated for 5 days at 20°C. Colonies with identical morphotypes were picked and purified by two additional rounds of re-streaking on YM agar and incubated at 20°C. The species was identified by ITS sequencing using ITS1F/ITS4 primers (3, 4) (accession number OZ507270.1). Sequence identity was 100% (406 nt overlap) to the *K. fluxuum* type strain NRRL YB-4273^T^ (KY103903).

Genomic DNA was isolated using a MasterPure Yeast DNA Purification Kit (Cambio) following the manufacturer’s instructions with additional zymolyase and proteinase K treatment steps from a 5-day liquid YM culture grown without agitation at 20°C. DNA concentration and purity were assessed using a Qubit 4.0 fluorometer (Thermo Fisher Scientific) and a NanoDrop 1000 spectrophotometer (Thermo Fisher Scientific). A short-read library was prepared using a Nextera DNA Flex library prep kit (Illumina) with DNA normalized to 5 ng/μL using a 20-fold diluted Illumina DNA Prep kit protocol (5) and sequenced on an Illumina NextSeq 500, producing 12.47 million paired-end 150 bp reads. A Nanopore library was prepared from the same gDNA prep, without fragmentation or size selection, using the SQK-LSK109 1D ligation kit (Oxford Nanopore Technologies [ONT]), and sequenced on a MinION instrument (ONT) with a R9.4.1 flow cell (ONT), producing 499,066 reads. Base calling was performed using Guppy (v3.6.0, ONT) in high-accuracy mode (model dna_r9.4.1_450bps_hac).

Default parameters were used for all software unless otherwise specified. Illumina reads were filtered with fastp v0.23.2 (6). Nanopores were filtered using NanoFilt v2.3.0 (7), selecting reads with quality ≥10 and length ≥5,000 bp. After filtration, 105,328 Nanopore reads were retained (read N_50_ = 20,426 bp). *De novo* assembly of Nanopore reads was performed using Flye v2.9.1 (8), using long reads in “ONT regular reads, pre-Guppy5” mode. The assembly was polished with the Illumina data using three rounds of alignment with BWA-MEM v0.7.17.1 (9) and Pilon v1.24 (10) correction. The final assembly consists of 10 contigs, including 4 with a putative telomeric repeat sequence (5′-TTGGTTGGTGCGAG-3′)_n_ (11), identified using Tandem Repeats Finder v4.09 (12), at one end. The total length is 13,031,074 bp, with an N_50_ of 2,201,021 bp and a G + C content of 32.05%. Genome completeness was estimated at 98.4% using BUSCO v5.5.0 (13) with the saccharomycete_odb10 data set.

The NCYC 4492 assembly (GCA_987432755.1) has an average nucleotide identity (14) of 99.83% to the NRRL YB-4273^T^ assembly (GCA_003707275.3) (15). Augustus v3.2.3 (16) predicted 5,679 protein-coding genes using the *Saccharomyces cerevisiae* training data set.

## Data Availability

This whole-genome shotgun project has been deposited in DDJB/ENA/GenBank (BioProject number PRJEB123523, assembly accession number CFYARH010000000). The version described in this paper is version 1. The raw reads are available in the NCBI Sequence Read Archive under accession numbers ERR17691141 (ONT) and ERR17691143 (Illumina). The predicted protein data set has been deposited in Zenodo (https://doi.org/10.5281/zenodo.20664952).
